# Effectiveness of Chatbots in Mental Health Screening and Assessment: Systematic Review

**DOI:** 10.2196/93672

**Published:** 2026-10-06

**Authors:** Isabel Morales Gil, Inés Marti-Estevez, Sandra Doval, José Miguel Gutiérrez-Carrillo, Rocío Fausor, Silvia Arribas-García, Miguel Ángel Álvarez-Mon, Javier Quintero

**Affiliations:** 1 Universidad Internacional De La Rioja, Avenida de la Paz, Logroño, La Rioja, Spain, +34 941 20 97 43; 2 Hospital Infantil Universitario Niño Jesús, Madrid, Spain; 3 Gijón Hospital, Gijón, Spain; 4 Valencian International University, Valencia, Spain; 5 Department of Legal Medicine and Psychiatry, Complutense University, Madrid, Spain; 6 Department of Psychiatry, Infanta Leonor University Hospital, Madrid, Spain; 7 Ramón y Cajal Institute of Sanitary Research (IRYCIS), Ramón y Cajal Hospital, Madrid, Spain; 8 CIBERSAM-ISCIII (Biomedical Research Networking Centre in Mental Health), Madrid, Spain; 9 Foundation for Biomedical Research and Innovation Hospital Universitario Unfanta Leonor Sureste, Madrid, Spain

**Keywords:** chatbots, AI, large language models, mental health, psychological assessment, screening

## Abstract

**Background:**

Mental health disorders affect 970 million people globally; yet, over 50% do not access timely evaluation due to structural barriers and professional shortages. Chatbots and AI-based conversational agents have emerged as promising tools for mental health screening and assessment.

**Objective:**

This study systematically evaluated the effectiveness, accuracy, reliability, and acceptability of chatbots and AI-based conversational agents for mental health screening and assessment in adults.

**Methods:**

Systematic search conducted in May 2025 across PubMed/MEDLINE, PsycINFO, Scopus, and Web of Science (2019‐2025), following PRISMA (Preferred Reporting Items for Systematic Reviews and Meta-Analyses) 2020 guidelines. Eligible studies evaluated chatbots or AI for mental health screening/assessment in adults (≥18 y). Risk of bias was assessed using appropriate tools (Risk of Bias 2, Quality Assessment of Diagnostic Accuracy Studies-2, Joanna Briggs Institute checklists, and Mixed Methods Appraisal Tool). This systematic review was registered with PROSPERO (International Prospective Register of Systematic Reviews; CRD420251072392).

**Results:**

Eighteen studies (2021‐2025) were included, with samples ranging from 20 to 3902 participants. Rule-based chatbots demonstrated high reliability (Cronbach α >0.85) and good acceptability (Acceptability of Intervention Measure >19/25). Generative models (large language models) achieved sensitivities of 0.84 to 0.93 and specificities of 0.80 to 0.96 for depression and anxiety, with correlations up to *r*=0.96 with expert clinicians in suicide risk assessment. Hybrid approaches combining large language models with machine learning achieved exceptional performance for cognitive impairment (*F*_1_-score of 92.1%, specificity of 99.6%). Most studies reported high user satisfaction (≥70%), although barriers existed among older populations. Methodological quality was heterogeneous with a moderate risk of bias in critical dimensions.

**Conclusions:**

Chatbots and AI conversational agents demonstrate clinically relevant performance in mental health screening and assessment. However, safe implementation requires clear clinical protocols, professional supervision, integration with electronic health records, and active mitigation of algorithmic bias. These technologies should complement rather than replace clinical judgment.

## Introduction

Mental health disorders constitute one of the leading causes of disability worldwide, affecting 970 million people in 2019, with depressive and anxiety disorders being the most prevalent diagnoses [1]. Globally, these conditions account for approximately 14% of disability-adjusted life years, with depression alone ranking as the third leading cause of disease burden [2]. In Spain, it is estimated that 1 in 4 adults will experience a mental health disorder throughout their lifetime, reflecting a significant burden on the health care system [3]. However, more than 50% of individuals with symptoms related to these disorders do not access timely evaluation due to structural barriers, social stigma, and a shortage of professionals [4]. Average waiting times for initial psychiatric assessment can exceed 60 days in many European health systems, with significant geographical disparities between urban and rural areas [5]. This treatment gap is particularly pronounced in low- and middle-income countries, where up to 85% of individuals with mental health needs receive no care at all [6].

This care gap, exacerbated by prolonged waiting lists and geographical inequalities, has driven the search for scalable digital solutions that can expand coverage and alleviate clinical care services. In this context, chatbots and conversational agents based on AI emerge as promising tools for psychological screening and assessment, offering continuous availability (24/7), replicating validated protocols, and automatically referring urgent cases to professionals [7,8]. Early systematic reviews demonstrated that conversational agents show promising efficacy and acceptability in mental health contexts, though most evidence focused on therapeutic interventions rather than assessment capabilities [9,10]. These systems can be broadly categorized into rule-based chatbots, which follow predetermined decision trees and administer standardized psychometric scales, and generative models based on large language models (LLMs) such as ChatGPT-4o (OpenAI), Claude 3.5 (Anthropic), and Gemini 1.5 (Google), which can engage in more flexible, context-aware conversations and interpret unstructured clinical narratives [11,12]. Rule-based systems, which dominated mental health chatbot research until 2023, offer advantages in transparency, clinical control, and consistent administration of validated instruments, with proven reliability for structured screening protocols [10,13]. However, they lack the flexibility to adapt to individual patient narratives or handle complex, nuanced clinical presentations [14]. Furthermore, the integration of prioritization algorithms and real-time data analysis enables the generation of more accurate and personalized assessments, aligning with the European Commission’s recommendations for the digitalization of mental health care [4].

The landscape shifted dramatically with the emergence of advanced LLMs from 2023 onward. Recent systematic analyses show that LLM-based chatbots surged from 16% of studies in 2022 to 45% in 2024, becoming the most frequently studied architecture in mental health AI research [15]. These models demonstrate capabilities that extend beyond simple symptom checklists: they can engage in open-ended clinical dialogue, interpret contextual information, generate psychodynamic formulations, and perform diagnostic reasoning that approaches the performance of experienced clinicians [8,11]. For instance, GPT-4 has passed psychiatric licensing examinations and demonstrated diagnostic accuracy comparable to psychiatrists in standardized clinical scenarios [16,17]. Recent evaluations of state-of-the-art models, including DeepSeek-R1 (DeepSeek), GPT-4.1 (OpenAI), and Llama 4 (Meta Platforms, Inc), have shown exceptional performance in both mental health knowledge assessment and illness diagnosis tasks [18].

In recent years, AI has demonstrated a disruptive role in mental health, with applications ranging from early symptom detection to the automation of therapeutic interventions. Recent reviews indicate that conversational systems can accurately identify linguistic and behavioral patterns associated with depression and anxiety in screening contexts, with high precision rates [7,19]. Beyond depression and anxiety, emerging evidence suggests potential applications in detecting cognitive impairment [12,20], assessing suicide risk [21,22], evaluating thought disorder in schizophrenia [23,24], and screening for postpartum posttraumatic stress disorder (PTSD) [25]. A detailed scoping review of 95 studies found that 71% of LLM applications in mental health focused on screening and detection tasks, with reported accuracies ranging from 0.80 to 0.96 for depression and anxiety classification [7]. In suicide risk assessment, LLMs have demonstrated correlations with expert clinicians exceeding *r*=0.90 in standardized vignette studies; however, concerns persist about the potential underestimation of risk in complex cases [26,27]. These technologies not only expand access but also facilitate personalized, scalable, and cost-effective interventions, which are critical given the growing demand and shortage of human resources.

However, their implementation raises ethical and methodological challenges: the need for clinical supervision, protection of sensitive data, algorithmic transparency, and the absence of longitudinal studies evaluating their sustained impact [28,29]. Of particular concern is algorithmic bias, which can arise across the AI life cycle and threaten equity in AI-assisted mental health assessment [28]. Systematic investigations have revealed that AI tools for mental health screening demonstrate differential performance across demographic groups, with sensed-behavioral patterns showing inconsistent relationships with depression symptoms across age, race, and socioeconomic subgroups [30]. Qualitative analyses of leading LLMs found that race-explicit or race-implied patient information frequently resulted in inferior treatment recommendations, though diagnostic decisions showed less bias [7]. Natural language processing models trained on public datasets exhibit measurable biases related to religion, race, gender, nationality, sexuality, and age in their mental health terminology [31,32]. These biases can perpetuate health disparities and undermine the equity of AI-powered mental health tools, particularly affecting marginalized populations who already experience barriers to quality care [28,33]. Moreover, current evidence is heterogeneous and limited, focused primarily on acceptability and user experience, while key aspects such as diagnostic validity, reliability, clinical utility, and equity in access have been less explored.

Previous systematic reviews have examined AI applications in mental health interventions and user acceptability [7,19], but these studies largely predate the emergence of advanced generative models and do not specifically focus on assessment capabilities. Despite growing interest in the use of chatbots and AI systems for psychological assessment, the available evidence lacks a critical synthesis integrating performance metrics, acceptability, and clinical applicability in the context of recent advances in generative models. This systematic review seeks to fill that gap by evaluating the effectiveness, accuracy, reliability, and acceptability of these tools, with the aim of guiding their safe and evidence-based implementation in health care settings.

## Methods

### Protocol Registration

This systematic review was registered in PROSPERO (International Prospective Register of Systematic Reviews) on August 2, 2025 (registration number CRD420251072392). The review followed the PRISMA (Preferred Reporting Items for Systematic Reviews and Meta-Analyses) 2020 guidelines and was structured according to the PIO (Population, Intervention, Outcome) framework; the completed PRISMA 2020 checklist is provided in Checklist 1.

.

Multimedia Appendix 1

### Eligibility Criteria

Studies were selected according to predefined criteria aligned with the research question and the PIO structure:

#### Inclusion Criteria

Inclusion criteria were as follows:

- Publications between 2019 and 2025 in English or Spanish.
- Adults (≥18 y) with a diagnosed or suspected psychological or psychiatric disorder.
- Evaluation of chatbot or AI use for psychological screening or assessment.
- Original peer-reviewed studies: randomized controlled trials (RCTs), validation studies, diagnostic accuracy studies, observational studies, qualitative studies, mixed methods studies, and pilot and feasibility studies.

#### Exclusion Criteria

Exclusion criteria were as follows:

- Studies focusing exclusively on therapeutic intervention.
- General medical care without a psychological assessment component.
- Conference abstracts without full text, editorials, opinion pieces.
- Narrative reviews (systematic reviews included only for context).
- Pediatric or adolescent population (<18 y).

### Information Sources and Search Strategy

The bibliographic search was conducted in May 2025 in the following electronic databases: PubMed/MEDLINE, PsycINFO, Scopus, and Web of Science Core Collection. These sources were selected for their relevance to biomedical, psychological, and multidisciplinary fields, ensuring comprehensive coverage of studies on AI and mental health.

For each database, specific search strategies were designed, combining controlled vocabulary terms (MeSH in PubMed and the Thesaurus in PsycINFO) and free-text keywords, adapted to the syntax and Boolean operators of each platform.

- Population: adults with mental disorders or psychological symptoms;
- Intervention: chatbots, conversational agents, AI applied to psychological assessment;
- Outcomes: effectiveness, diagnostic accuracy, reliability, validity, acceptability, and feasibility.

The following limits were applied: publications between 2019 and 2025, in English or Spanish. No study design restrictions were applied in the initial search. Additionally, the reference lists of included studies and relevant systematic reviews were manually reviewed to identify additional literature (Table 1).

**Table 1.** Characteristics and results of included studies.

Authors (y)	Sample/data (N)	Chatbot/AI	Condition assessed	Design and reference instrument(s)	Key findings
Anmella et al [34], 2023	34 (primary care and health care workers)	Vickybot	Anxiety, depression, work-related burnout, and suicidal risk	Multiphase development plus feasibility and potential effectiveness study: single-arm longitudinal study (1 mo follow-up) with self-assessments at baseline, 2 weeks, and 4 weeks PHQ-9^a^; GAD-7^b^; MBI^c^	After 2 weeks of use, no significant differences for anxiety or depressive symptoms; burnout moderately reduced (*z*=−2.07, *P*=.04), 9% activated suicide alert
Bartal et al [25], 2024	1295 (postpartum)	ChatGPT-3.5-turbo-16k+embeddings ADA^d^+DNN^e^	Postpartum PTSD^f^	Cross-sectional research Maternal narratives and PCL-5^g^	ChatGPT-3.5‐16k zero-shots AUC^h^=0.60, *F*_1_-score=0.33, sensitivity=0.20, specificity=0.99;/few-shots AUC=0.60, *F*_1_-score=0.38, sensitivity=0.24, specificity=0.96; model embeddings AUC=0.80, *F*_1_-score=0.81, sensitivity=0.85, specificity=0.75
de Arriba-Pérez et al [20], 2024	30 (57% with cognitive impairment)	Celia (chatbot+ML^i^ with feature extraction using GPT LLM^j^)	Cognitive impairment	Cross-sectional research Clinical diagnosis	LLM-features+RF^k^: accuracy=98.47%, sensitivity (impairment)=97.78%; >n grams (76.67%) and >direct ChatGPT (57%‐61%)
Dosovitsky et al [35], 2021	3895 adults over 65 years	Tess (chatbot)	Depression	Cross-sectional research A chatbot version of PHQ-9	α=0.896
Du et al [12], 2024	Clinical EHRs^l^: 4949 sections for baseline models and 1996 sections for final testing	Llama 2 vs GPT-4+DNN+XGBoost^m^ ensemble	Cognitive impairment	Cross-sectional comparative study Clinical diagnosis in EHR	Ensemble models are better than individuals. Ensemble *F*_1_-score=92.2%; recall=94.2%; precision=90.3%; E=99.6%
He et al [36], 2024	100 web-based medical consultation samples, with 239 randomly selected consultation questions	ChatGPT-4 vs ERNIE bot 2.2.3	Autism	Cross-sectional research Comparative assessment (physicians vs 2 AI chatbots); Clinical diagnosis	Physicians scored higher in relevance (H=111.67, *P*<.001; MD^n^=3.75, 95% CI 3.63‐3.82) and usefulness (H=135.81, *P*<.001; MD=3.54, 95% CI 3.47‐3.62); ChatGPT-4 scored higher in empathy (H=118.58, *P*<.001; MD=3.64, 95% CI 3.57‐3.71); no differences between physicians and ChatGPT 4 for correctness (H=49.99, *P*<.001); ERNIE obtained the lowest scores across all 4 dimensions
Hur et al [37], 2024	467	ChatGPT-3.5/4; Linguistic Inquiry and Word Count (LIWC-22)	Depression (prognosis)	Longitudinal (3-wk follow-up) Open-ended questions; PHQ-9	Human-rated sentiment score predicted depression trajectory in individuals with minimal symptoms (β=−0.15^o^, SE=0.05, *t*=−2.95^p^, *P*=.003) or with mild-to-moderate symptoms (β=−0.29, SE=0.09, *t*=−3.23, *P*=.001). ChatGPT also predicted future depressive symptoms in a similar way to humans; LIWC did not predict prognosis
Kang and Hong [38], 2025	20	HoMemeTown Dr CareSam vs Woebot/Happify	Primary (suicidal ideation and severe depression) and secondary (sleep disturbances and social withdrawal) risk indicators+seamless support	Pilot testing of the chatbot Cross-sectional research One overall satisfaction question and 7 quantitative items assessing effective psychological counseling	High satisfaction: support (mean 9.0, SD 1.2); perceived empathy (mean 8.7, SD 1.6); and active listening (mean 8.0, SD 1.8). Satisfaction level higher vs Woebot and Happify (*F*=12.94, *P*<.001)
Kaywan et al [39], 2023	50	DEPRA (depression analysis chatbot, based on the Structured Interview Guide for the HDRS^q^ [SIGH_D])	Depression (early detection)	Nonclinical trial IDS (Inventory of Depressive Symptomatology); QIDS (Quick Inventory of Depressive Symptomatology)+user satisfaction	Overall satisfaction: 79% scored 3.95/5 both scoring returned to a similar outcome with slight variations for depression level
Kosyluk et al [40], 2024	Of the 329 participants, only 222 (67.5%) used the chatbot	Tabatha chatbot	Psychological distress	Pilot testing of the chatbot Cross-sectional research PHQ-9	Of the 222 individuals who used the chatbot, 168 (75.7%) completed the PHQ-9 screening and 164 (73.9%) completed the acceptability
Lho et al [41], 2025	1064	General-purpose LLMs (GPT-4o, GPT-3.5-turbo-16k, and Gemini 1.0 Pro) and text-embedding models (OpenAI text-embedding-3-large, -3-small, and ada-002)	Clinically significant depression and high suicide risk	Cross-sectional, retrospective observational study using SCT^r^ narratives and self-report scales K-BDI-II^s^, Zung Self-Rating Depression Scale, Beck Scale for Suicidal Ideation, and K-WAIS-IV^t^ (FSIQ^u^)	LLMs and embedding-based models achieved AUROC^v^>0.7 for detecting depression and suicide risk, with best performance for self-concept narratives and text-embedding-3-large+XGB (AUROC 0.84 for depression)
Li et al [8], 2024	20	GPT-4	Adult-acquired buried penis	Qualitative description study (semistructured interviews) None (qualitative analysis of transcripts)	GPT-4 identified key themes (urinary/sexual/mental health issues) with moderate agreement to humans (κ=0.401); humans found richer subthemes; AI consistent across iterations
Liu et al [42], 2025	200	ChatGPT-4 (generating GPT-PHQ-9 and GPT-GAD-7)	Anxiety and depression	Cross-sectional study PHQ-9 and GAD-7	GPT-PHQ-9 and GPT-GAD-7 demonstrated good agreement (ICC^w^ 0.80/0.70, ρ 0.63/0.68, AUC 0.96/0.86) with optimal cutoffs of 9.5 and 6.5, respectively
McBain et al [21], 2025	N/A^x^ (24 hypothetical scenarios; no human participants)	ChatGPT-4o, Claude 3.5 Sonnet, and Gemini 1.5 Pro	Suicidal ideation	Observational cross-sectional study SIRI-2 (Suicidal Ideation Response Inventory–revised)	LLMs showed upward bias rating responses as more appropriate vs expert suicidologists (MD 0.61‐0.86); SIRI-2 scores: ChatGPT 45.7 (≈master’s counselors), Claude 36.7 (>trained mental health professionals), and Gemini 54.5 (≈untrained school staff)
Ohse et al [43], 2024	51	GPT-4 (LLM model, zero-shot, and no fine-tuning)	Social anxiety (Social Anxiety Disorder [SAD])	Cross-sectional, semistructured interviews based on LSAS^y^ with transcript analysis. Social Phobia Inventory (SPIN, German version), cut-off 25	High correlation (*r*=0.79) between GPT-4 predictions and actual SPIN; *F*_1_-score accuracy=0.84 (threshold 25); AUC=0.93; optimal threshold 18
Shin et al [44], 2024	91	GPT-3.5 and GPT-4 (con/sin fine-tuning and chain-of-thought prompting)	Depression	Retrospective analysis of 2-week diary writing via app (EMA^z^ text data) PHQ-9, cut-off ≥10 Beck Scale for Suicide Ideation (cut-off ≥8); clinical review by psychiatrists	GPT-3.5 fine-tuning: accuracy 0.902, specificity 0.955; GPT-3.5 without fine-tuning: balanced accuracy 0.844, recall 0.929; useful diary entries for depression screening
Shinan-Altman et al [22], 2024	No human participants; 160 AI evaluations (10 per vignette across 8 vignettes for each of ChatGPT-3.5 and ChatGPT-4)	ChatGPT-3.5 and ChatGPT-4	Suicide risk (suicidal thoughts, attempts, serious attempts, and mortality), influenced by history of depression and access to weapons	Experimental vignette study with multivariate 3-way ANOVA (factors: depression history, weapon access, and gender) and Bonferroni post hoc tests Custom Likert-scale questions (0‐7: very slight to very high likelihood) adapted from Levi-Belz and Gamliel [45]	Both models recognized depression history as a risk factor; ChatGPT-4 showed nuanced integration of weapon access and interaction effects, assigning higher severity ratings overall vs ChatGPT-3.5.
So et al [46], 2024	10	GPT-4 Turbo and GPT-3.5 Turbo (zero-shot, few-shot, fine-tuning, and RAG^aa^)	PTSD symptoms, depressive/anxiety disorders, and alcohol use disorder	Pilot study: LLM alignment on labeled transcripts (training/validation/test split) Metrics include recall midtoken distance, *F*_1_-score, G-Eval, and BERT^ab^ Score.	LLMs achieved >0.8 accuracy/*F*_1_-score for symptom naming (fine-tuning best); 70% segments with *d*≤20 for section delineation; high G-Eval (>4.6 coherence) for summaries using stressors+symptoms
Voppel et al [23], 2021	100 (50 Schizophrenia-spectrum disorders patients+50 controls)	word2vec semantic model	Schizophrenia-spectrum disorders	Semistructured interview; word2vec analysis; and RF classification PANSS^ac^; CASH^ad^/MINI^ae^	85% classification accuracy (86% sensitivity and 84% specificity)

a PHQ-9: Patient Health Questionnaire-9.

b GAD-7: Generalized Anxiety Disorder-7.

c MBI: Maslach Burnout Inventory.

d ADA: assessment of diagnostic accuracy.

e DNN: deep neural network.

f PTSD: posttraumatic stress disorder.

g PCL-5: PTSD Checklist for* DSM-5*.

h AUC: area under the curve.

i ML: machine learning.

j LLM: large language model.

k RF: random forest.

l EHR: electronic health record.

m XGBoost: Extreme Gradient Boosting.

n MD: mean difference.

o Beta (β) coefficient from robust linear regression.

p *t* statistic associated with the estimated regression coefficient in the robust linear regression model.

q HDRS: Hamilton Depression Rating Scale.

r SCT: sentence completion test.

s K-BDI-II: Korean Beck Depression Inventory–Second Edition.

t K-WAIS-IV: Korean Wechsler Adult Intelligence Scale–Fourth Edition.

u FSIQ: Full-Scale IQ.

v AUROC: area under the receiver operating characteristic curve.

w ICC: intraclass correlation coefficient.

x N/A: not applicable.

y LSAS: Liebowitz Social Anxiety Scale.

z EMA: ecological momentary assessment.

aa RAG: retrieval-augmented generation.

ab BERT: Bidirectional Encoder Representations from Transformers.

ac PANSS: Positive and Negative Syndrome Scale.

ad CASH: Comprehensive Assessment of Symptoms and History.

ae MINI: Mini-International Neuropsychiatric Interview.

### Study Selection

The selection process was carried out in two phases: (1) initial screening of titles and abstracts and (2) full-text evaluation to determine final eligibility.

Three independent reviewers conducted the screening in parallel (JMG-C, IM-E, and IMG), using the bibliographic management tool Rayyan. Discrepancies were resolved through discussion and, when necessary, with the intervention of a fourth reviewer to reach consensus.

The previously defined inclusion and exclusion criteria (*Eligibility Criteria*) were applied. The complete process was documented following the PRISMA 2020 guideline and is presented in the *Results* section (PRISMA diagram), which details the number of identified records, those eliminated due to duplication, those excluded after title/abstract review, and the reasons for exclusion in the full-text phase.

### Data Extraction and Quality Assessment

Data extraction was performed independently by 2 reviewers, using a standardized template designed for this review. For each included study, the following variables were collected:

- General information: author, year of publication, country, and language.
- Population characteristics: sample size, inclusion criteria, and diagnosis or condition evaluated.
- Intervention: type of chatbot or AI system (model, platform, and architecture), interaction modality (text and voice), and context of use (clinical, community, and experimental).
- Reference instruments: psychometric scales, diagnostic interviews, or a gold standard used for comparison.
- Main outcomes: performance metrics (sensitivity, specificity, area under the curve [AUC], *F*_1_-score, and intraclass correlation coefficient), validity and reliability indicators, acceptability, usability, and feasibility.
  - In case of discrepancies between reviewers, these were resolved by consensus or with the intervention of a third evaluator.
- Risk of bias assessment.
  - The risk of bias was assessed using specific tools according to the methodological design of each study:
- RCTs: Cochrane Risk of Bias 2 (RoB 2) tool.
- Diagnostic accuracy studies: Quality Assessment of Diagnostic Accuracy Studies-2 (QUADAS-2) tool.
- Cross-sectional and cohort observational studies: Joanna Briggs Institute (JBI) checklist for analytical studies.
- Qualitative studies: JBI checklist for qualitative research.
- Mixed methods studies: Mixed Methods Appraisal Tool (MMAT).

Each study was classified into categories of low risk, moderate risk, or high risk in the evaluated domains. The results were graphically synthesized using stacked bar charts to facilitate the global interpretation of methodological quality.

## Results

### Study Selection

The initial search identified 583 records in the selected databases. After removing 99 duplicates, 484 titles and abstracts were evaluated, of which 412 were excluded for not meeting the inclusion criteria. Thirty-two full texts were reviewed, excluding 14 studies for the following reasons:

- Noneligible study type (n=10).
- Diagnosis unrelated to the research question (n=4).

Finally, 18 studies met the criteria and were included in the qualitative synthesis. The complete process is shown in the PRISMA diagram (Figure 1).

**Figure 1.**
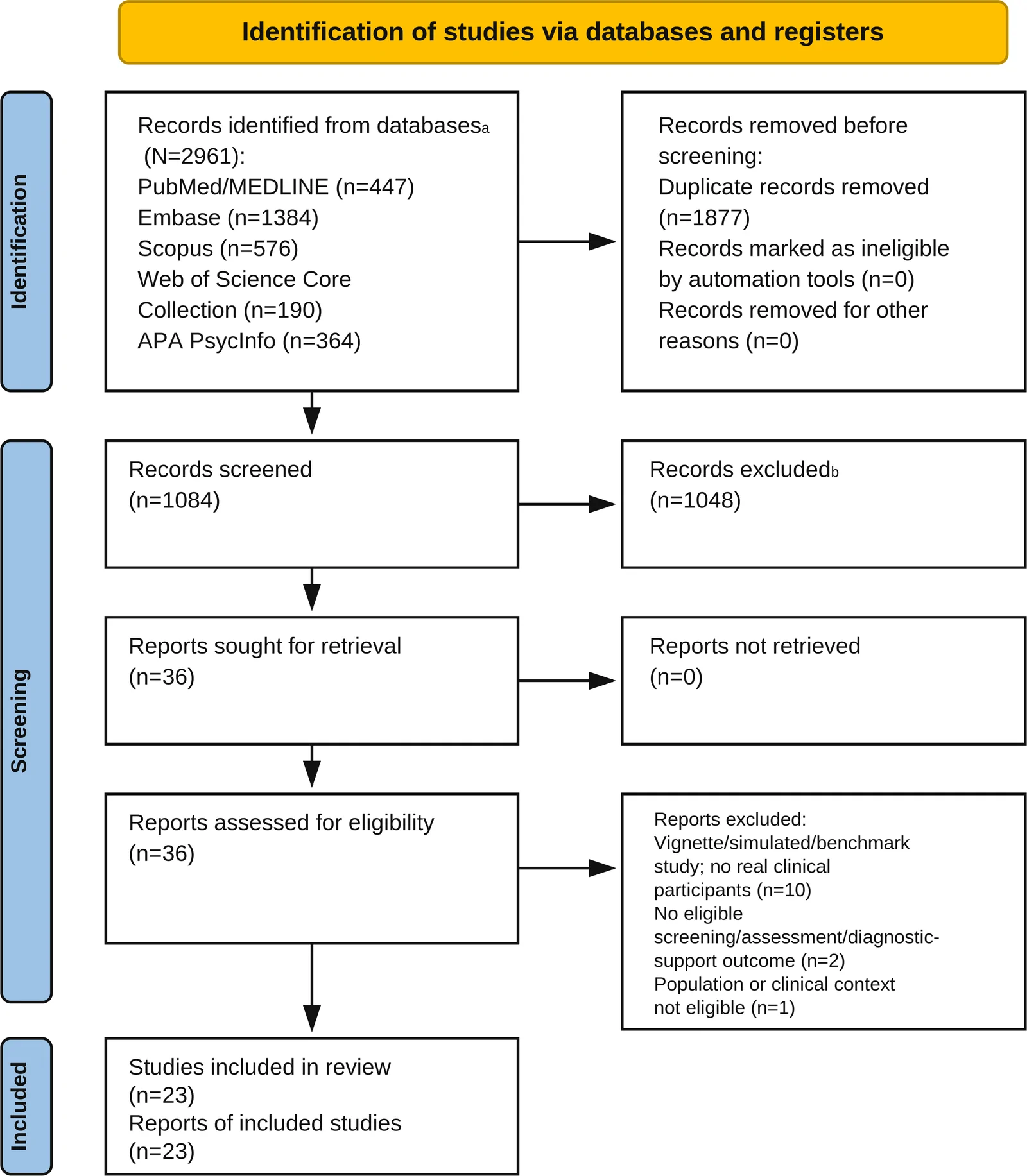
Study flowchart (produced according to the PRISMA [Preferred Reporting Items for Systematic Reviews and Meta-Analyses] 2020 flow diagram). ^a^Consider, if feasible, reporting the number of records identified from each database or register searched (rather than the total number across all databases/registers). ^b^If automation tools were used, indicate how many records were excluded by a human and how many were excluded by automation tools.

### Study Characteristics

The 18 included studies were published between 2021 and 2025, covering diverse geographical contexts (North America, Europe, Asia, and Oceania) and samples ranging from 20 to 3902 participants. Most studies were conducted in clinical, community, or university settings, with adult populations presenting with depressive disorders, anxiety, suicide risk, cognitive impairment, schizophrenia, or PTSD.

Regarding interventions, 2 major categories were identified:

- Rule-based chatbots are designed to administer psychometric scales or screening protocols.
- Generative models (LLMs), primarily ChatGPT (versions 3.5, 4, and 4o), along with other models such as Claude, Gemini, Llama, and commercial chatbots (eg, Woebot and Vickybot).

The most commonly used reference instruments were the Patient Health Questionnaire-9 (PHQ-9), Generalized Anxiety Disorder-7 Scale (GAD-7), Social Phobia Inventory (SPIN), suicide risk scales, including the Suicidal Ideation Response Inventory-2 (SIRI-2), structured diagnostic interviews, and cognitive tests. Methodological designs included RCTs, validation studies, cross-sectional studies, qualitative studies, and feasibility pilots, reflecting notable heterogeneity in objectives and metrics.

### Outcomes

The findings were grouped into 3 dimensions:

#### Type of Chatbot and Assessment Modality

On the one hand, rule-based chatbots showed high reliability in administering psychometric scales (Cronbach α >0.85) and good acceptability (Acceptability of Intervention Measure [AIM] scores >19/25). On the other hand, generative models (LLMs) achieved high diagnostic accuracy metrics in screening and classification tasks, with sensitivities between 0.84 and 0.93 and specificities between 0.80 and 0.96 for depression and anxiety (Figure 2 [12,20,23,25,44]).

**Figure 2.**
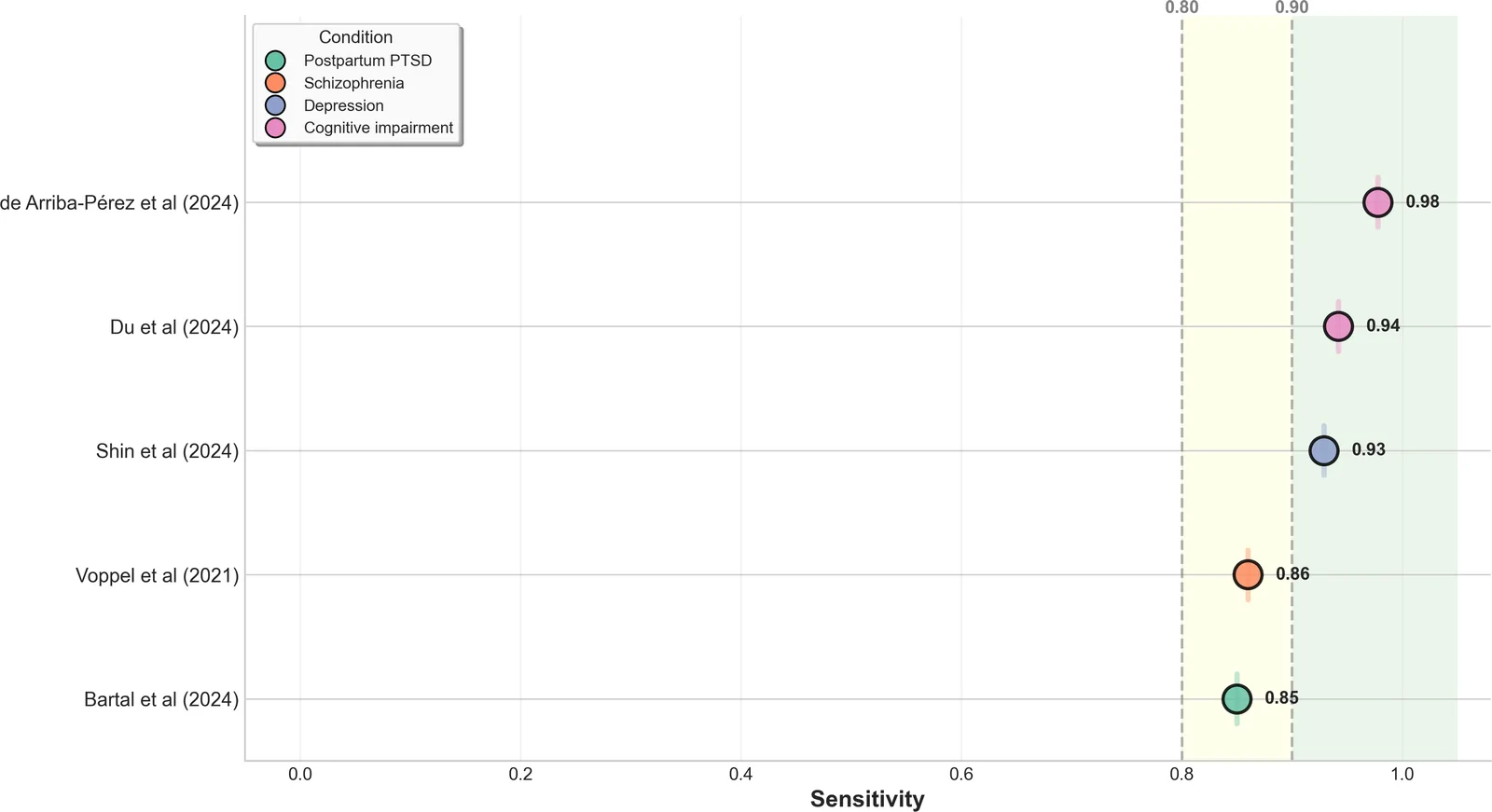
Forest plot of sensitivity (S) by study [12,20,23,25,44]. PTSD: posttraumatic stress disorder.

#### Type of Assessment (Screening vs Diagnosis)

Regarding screening, chatbots achieved equivalence with traditional methods on scales such as the PHQ-9 and GAD-7, with intraclass correlation coefficients of 0.70 to 0.80. In diagnosis tasks, LLMs showed performance comparable to professionals in complex tasks (eg, suicide risk and schizophrenia), with correlations with experts up to *r*=0.96 (Figure 3 [12,23,25,44]).

**Figure 3.**
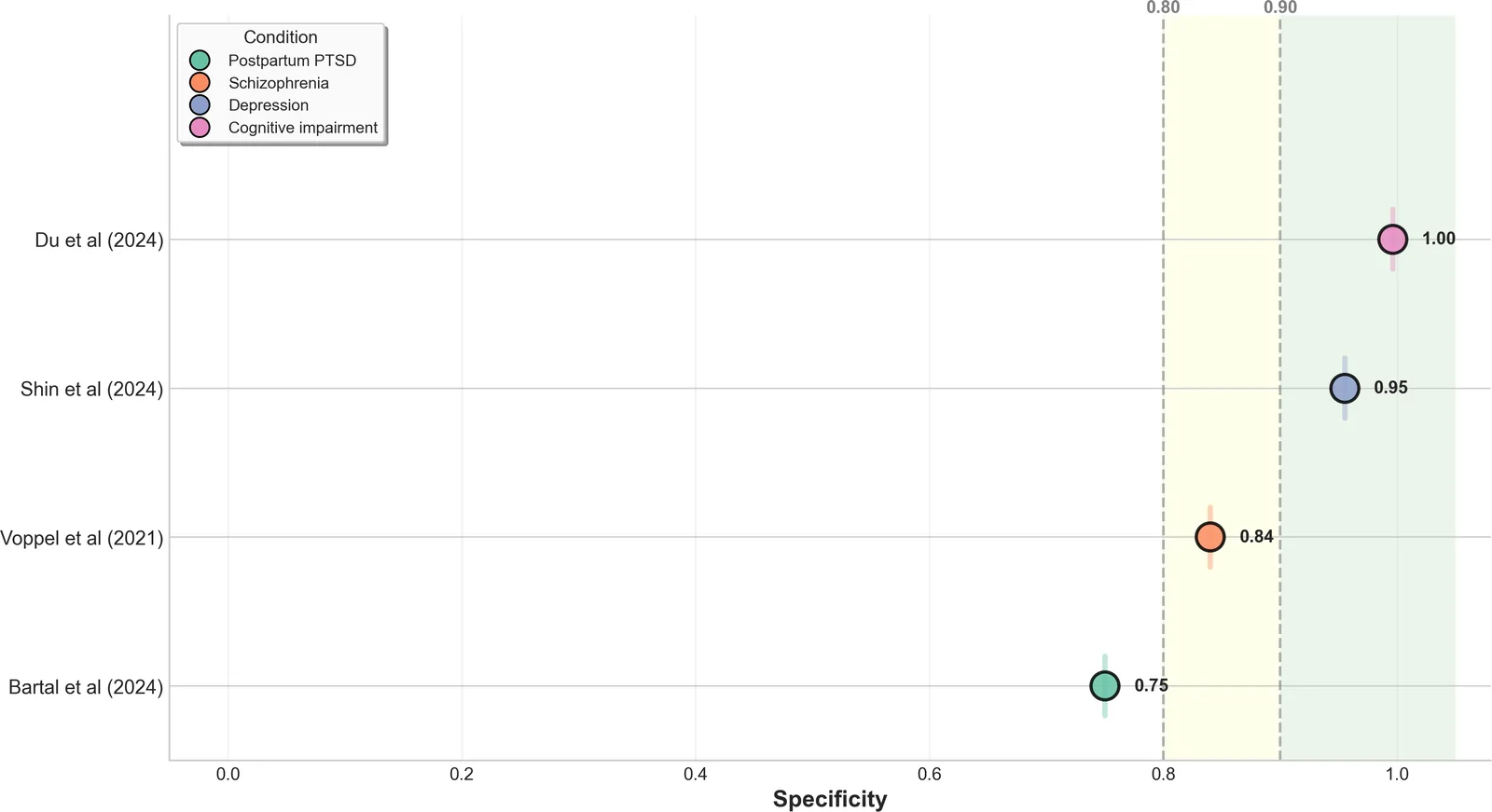
Forest plot of specificity (E) by study [12,23,25,44]. PTSD: posttraumatic stress disorder.

#### Type of Disorder

The greatest volume of evidence was found for depression and anxiety, with an AUC between 0.80 and 0.84 in adjusted models. Suicide risk assessment demonstrated a high concordance with experts (*r*>0.90) in clinical vignettes. Promising results were found for cognitive impairment and schizophrenia, especially when combining LLMs with machine learning (ML) techniques (*F*_1_-score >0.90 in some studies; Figure 4 [25,41]).

**Figure 4.**
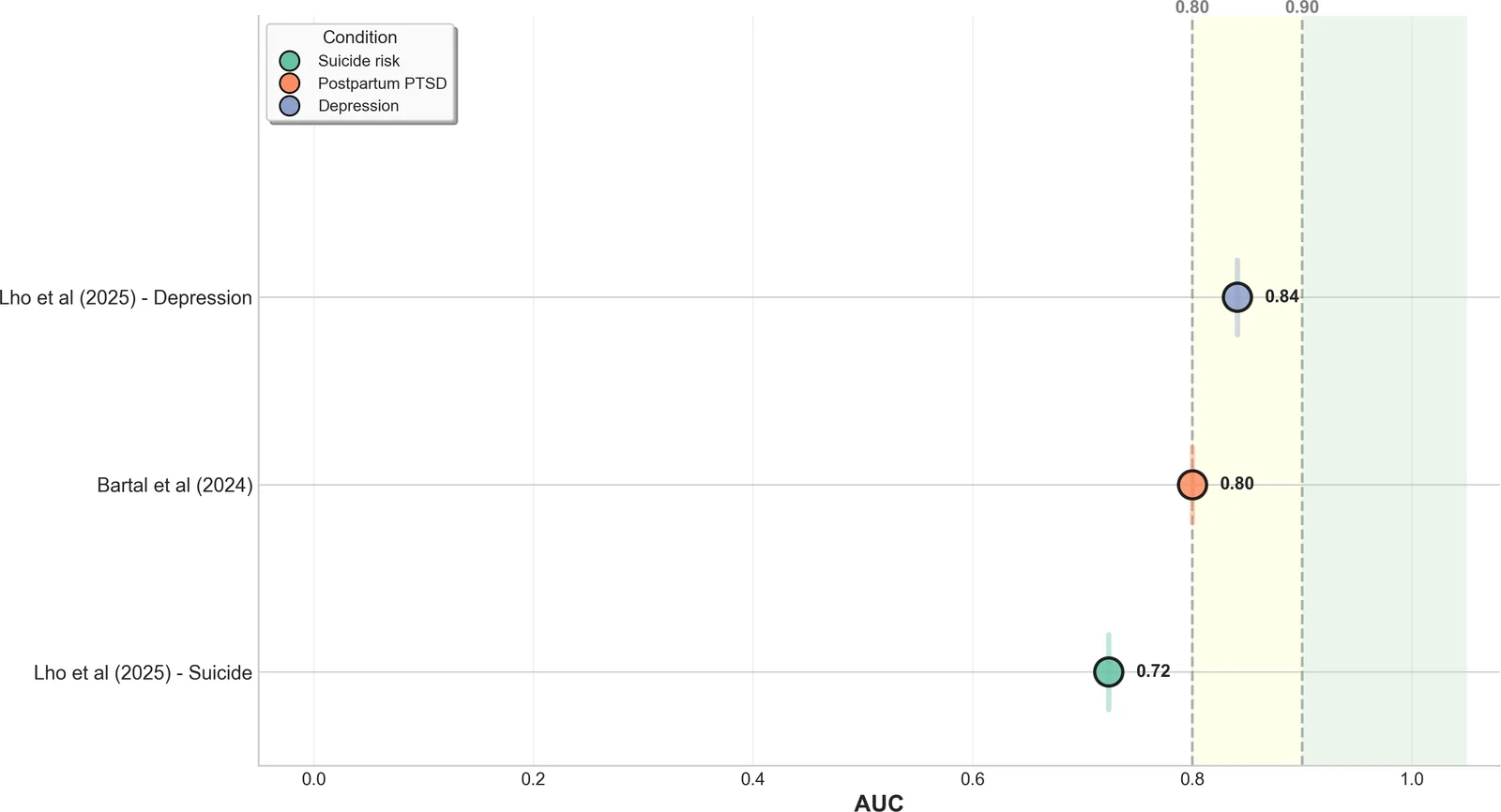
Forest plot of area under the curve (AUC) by study [25,41]. PTSD: posttraumatic stress disorder.

#### Acceptability and Usability

Most studies reported high satisfaction (≥70% of users) and good intention for sustained use, although barriers related to cognitive load and technological familiarity were identified in older populations.

Forest plots were created for sensitivity (S), specificity (E), AUC, *F*_1_-score, and accuracy, based on point estimates reported in the included studies. Since most articles did not report CIs or study weights, the figures show normalized point values (0‐1) with reference lines at 0.80 and 0.90. Overall, the results indicate high performance in language-based screening tasks (eg, depression, anxiety, and suicide risk), and high accuracies in hybrid approaches (LLM/embeddings+ML) for cognitive impairment; however, variability between conditions and designs is observed, which warrants cautious interpretations and the need for standardized reporting (metrics with CI and weighting by sample size).

### Use of a Chatbot for Diagnosis/Assessment (Through Direct Interaction)

#### Cognitive Impairment in Free Dialogue

In a conversational application, de Arriba-Pérez et al [20] compared 3 approaches: n-grams, a language model as a direct classifier, and a hybrid flow in which the model extracts language representations (features) and a random forest performs the classification. The hybrid flow achieved an accuracy of 98.47%, with macro averages close to 98% and sensitivity for the “impairment” class of 97.78%, clearly surpassing n-grams (76.67%) and the direct classifier (57.17%‐61.19%). These data show that conversational interaction combined with task-trained processing achieves very high performance (Figure 5 [12,25,43,47]).

**Figure 5.**
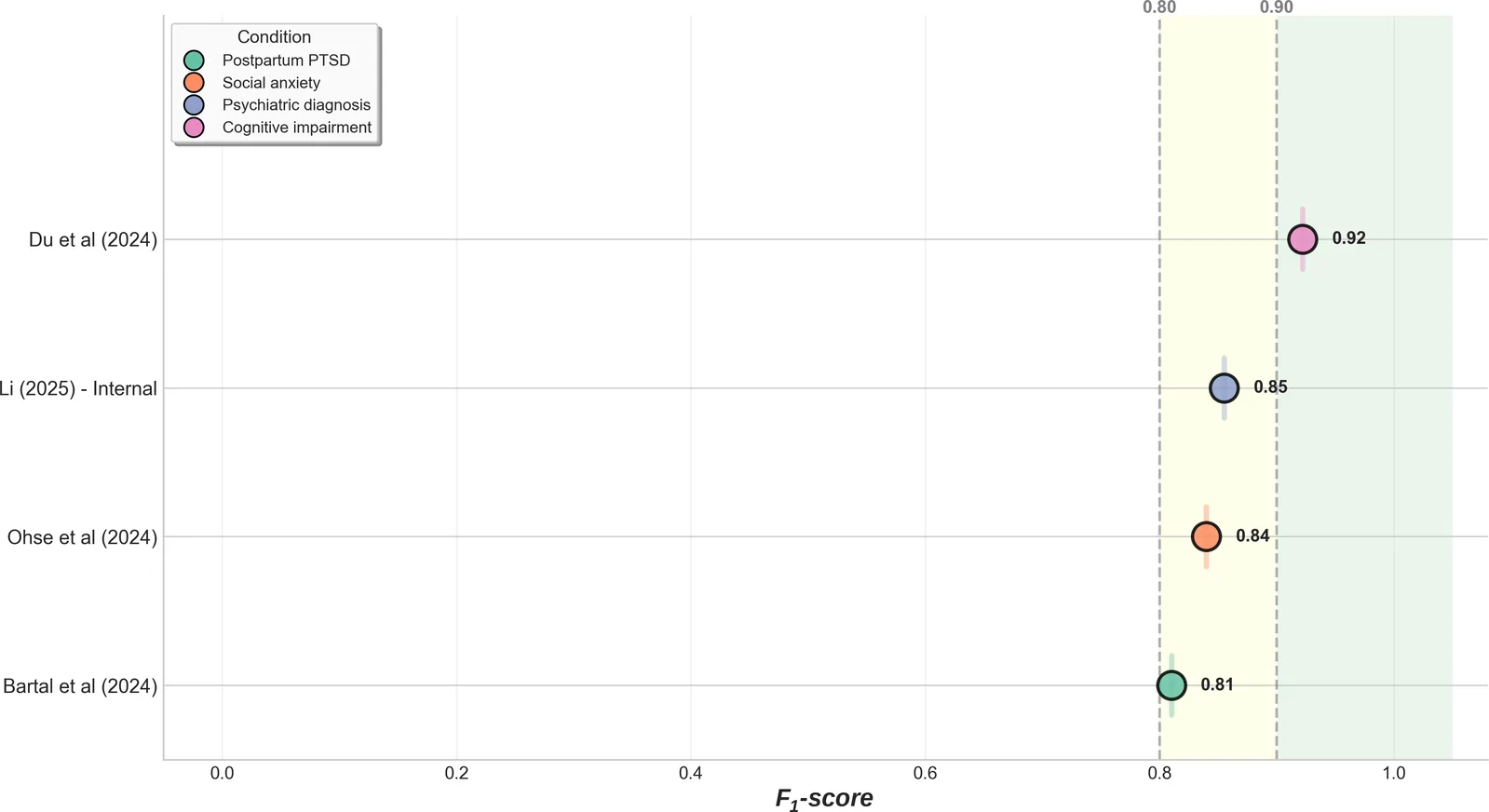
Forest plot of *F*_1_-score by study [12,25,43,47]. PTSD: posttraumatic stress disorder.

#### Chatbot-Guided Interview Combined With Neuroimaging

In a multimodal approach, Li [47] integrated a ChatGPT-guided interview with functional magnetic resonance imaging (fMRI) to classify psychiatric diagnoses in adults with a confirmed single diagnosis. The complete model achieved an accuracy of 85.7% in an internal test and 83.5% in external validation (n=100), with an *F*_1_-score of 85.5%. Ablation analyzes showed that the linguistic component alone reaches approximately 83% and that combining it with fMRI raises performance to approximately 87%. Compared with the study by de Arriba-Pérez et al [20], the result is somewhat lower, consistent with the greater complexity of multidiagnostic and multimodal classification.

In binary dialogue-based evaluation (impairment yes/no), figures close to 98% are reached [20]; in multidiagnostic classification guided by conversation and combined with neuroimaging, accuracy is around 85% to 84% [47] (Figure 6 [20,23,44,47]).

**Figure 6.**
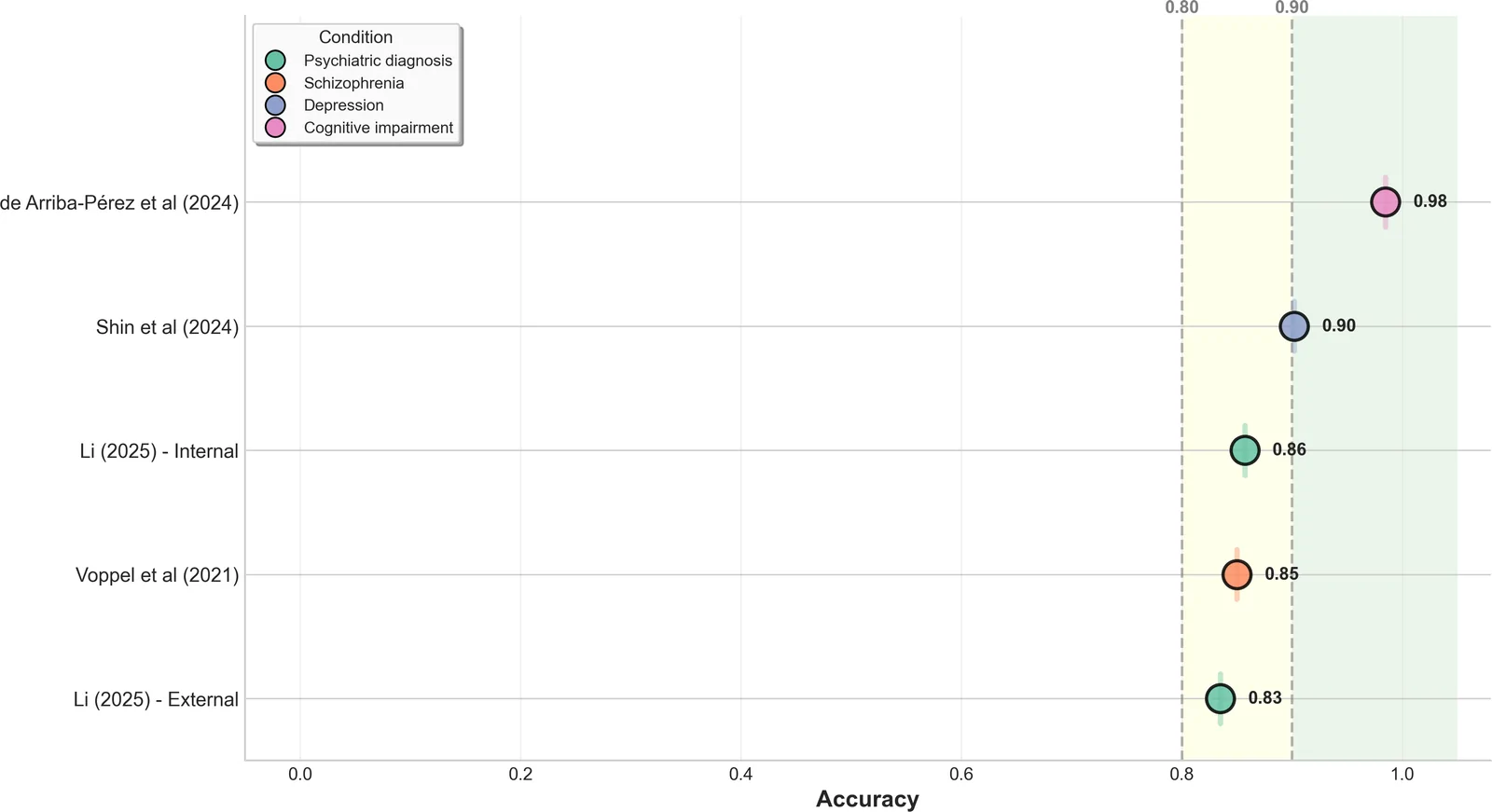
Forest plot of accuracy/precision by study [20,23,44,47]. PTSD: posttraumatic stress disorder.

### Use Language Models to Process Data (Without Direct Interaction)

#### Brief Clinical Text, Interviews, and Narratives

##### Depression and Suicide Risk in Short Clinical Narratives

In hospitalized patients, Lho et al [41] analyzed text from the incomplete sentences test. The model’s performance without examples was moderate (AUC 0.720 for depression; 0.731 for suicide risk), with improvement when providing a few examples for depression (0.754). The best result was offered by the combination of language representations+XGBoost, with AUC 0.841 (depression) and 0.724 (suicide); self-concept was the most informative textual domain.

##### Depression in Spontaneous Daily Writing

Shin et al [44] showed accuracy 0.902 and specificity 0.955 in personal diaries after model fine-tuning; without fine-tuning, the balanced accuracy reached 0.844 with sensitivity 0.929, supporting the use of ecological language for screening when the model is optimized.

##### Social Anxiety in Semistructured Interview

Ohse et al [43] found high convergent validity between model-estimated severity and SPIN (*r*=0.79) and a “probable” case classification with *F*_1_-score of 0.84. In clinical interview tasks, these figures are high.

##### Schizophrenia: Language and Thought

With semantic connectivity in the interview, Voppel et al [23] obtained an accuracy of 85%, sensitivity of 86%, and specificity of 84% (cross-validation). Pugh et al [24] showed that language models (GPT-3.5 or 4, Llama-3) match experts when scoring coherence, tangentiality, and content, although with variability between runs that improves when adjusting parameters and aggregating outputs. Overall, the linguistic signal for schizophrenia is robust when the method is stable.

##### Postpartum PTSD From Birth Narratives

Bartal et al [25] compared generative approaches with a classifier trained on language representations: AUC=0.80, *F*_1_-score=0.81, sensitivity=0.85, specificity=0.75, with clear superiority over “no fine-tuning” use. Compared to depression in diaries [44] and depression in clinical narratives [41], performance is similar (AUC≈0.80‐0.84).

##### Symptom Extraction and Clinical Summary

So et al [46] aligned a model to label symptoms in interviews, achieving *F*_1_-score of 0.82 and well-valued summaries in coherence and consistency. This support can structure interviews and save documentation time.

##### Multimodal Classification Based on Language+fMRI (Without Direct Conversation)

Li [47] showed that language alone already provides approximately 83% accuracy and that adding fMRI raises that value to approximately 87%, confirming the incremental value of combining text with biomarkers.

##### Electronic Health Records

Detection of cognitive impairment in electronic health records (EHRs; without keyword filters).

Du et al [12] developed an ensemble (generative model+attention neural network+Extreme Gradient Boosting [XGBoost]) that achieved *F*_1_-score of 92.1%, sensitivity of 94.2%, precision of 90.2%, and specificity of 99.6%, surpassing each component separately (the generative model, optimized by instructions, reached *F*_1_-score of 80.3%). Compared to studies with narratives or diaries, the EHR offers larger samples and, with the ensemble, very high metrics.

### Vignettes and Other Contexts

#### Judgment Before Suicidal Ideation (SIRI-2 Vignettes)

McBain et al [21] observed high correlations among suicidologists (*r*=0.96; 0.93; 0.81) and elevated reliability, although with a leniency bias; Shinan-Altman et al [22] showed that depression and access to weapons elevate risk assessment, a more consistent pattern in the more advanced version of the model.

#### Prediction of Symptom Changes

Hur et al [37] showed that linguistic sentiment modeled by a language model predicts worsening of depression at 3 weeks, with performance similar to human evaluators.

#### Other Comparative Areas

Jin et al [7] observed improvements when requesting explicit reasoning, with failures in atypical presentations; evidence indicates that AI tools for mental health screening may show differential performance across demographic subgroups, highlighting the need for systematic bias assessment [30].

### Risk of Bias

Risk of bias analysis was conducted applying specific tools according to the methodological design of each study. For RCTs, the Cochrane RoB 2 tool was used, while diagnostic accuracy studies were evaluated using QUADAS-2. Qualitative studies were analyzed with the JBI checklist for qualitative research, and mixed methods studies with the MMAT. Finally, cross-sectional studies were evaluated with the JBI checklist for descriptive studies. Each study was classified according to the risk of bias in the different methodological domains, assigning judgments of “low risk,” “moderate risk,” or “high risk.”

The results were visualized using stacked bar charts by domain, facilitating the global interpretation of the methodological quality of the included evidence. Cross-sectional studies presented a higher proportion of moderate bias in sampling strategies and measurement validity. In RCTs, the most frequent bias was observed in the blinding of participants and personnel. Studies evaluated with QUADAS-2 showed moderate bias in participant selection and intervention classification.

Additional per-study risk-of-bias visualizations are provided in Multimedia Appendix 2. Overall, the included studies present heterogeneous methodological quality, with a predominance of moderate bias in critical dimensions of internal validity. This methodological variability should be considered when interpreting the results of efficacy and accuracy of chatbots in psychological assessment.

## Discussion

### Principal Findings

This systematic review demonstrates that chatbots and AI-based conversational agents achieve clinically relevant performance in mental health screening and assessment. Rule-based systems showed high reliability (Cronbach α>0.85) in administering standardized scales, while generative LLMs achieved sensitivities of 0.84‐0.93 and specificities of 0.80‐0.96 for depression and anxiety. LLMs demonstrated strong correlations with expert clinicians (*r* values up to 0.96) in suicide risk assessment and exceptional performance when combined with ML (*F*_1_-score=92.1%) in cognitive impairment detection [12]. User acceptability was generally high (≥70%), although barriers existed for older adults and those with lower digital literacy. These findings suggest AI-driven tools can replicate traditional psychometric protocols while offering additional capabilities through natural language processing; however, high-performance metrics must be interpreted cautiously given methodological heterogeneity and moderate risk of bias.

Our findings extend beyond previous systematic reviews, whereas those reviews [9,10] reported promising but limited evidence for chatbot effectiveness in mental health contexts. These reviews focused primarily on therapeutic interventions rather than assessment capabilities. A critical distinction in our review is the emphasis on generative LLMs, which represent a qualitative advance from earlier rule-based systems. Our synthesis captures the rapid evolution from 2023 to 2025, during which LLM-based applications surged from 16% to 45% in mental health chatbot studies [15]. Recent evidence demonstrates that GPT-4 and similar models can pass psychiatric licensing examinations and perform diagnostic reasoning comparable to experienced clinicians [16,17], capabilities not observed in earlier generation systems. Our findings on cognitive impairment detection [12] and suicide risk assessment [21,27] extend the evidence base beyond the depression and anxiety focus of prior reviews.

### Strengths and Limitations of the Evidence Base

Included studies used appropriate reference standards, including validated instruments (PHQ-9 and GAD-7) and structured clinical interviews, with several using external validation cohorts. However, significant limitations imply careful interpretation. Risk of bias assessment revealed moderate to high risk in critical domains, particularly participant selection, blinding procedures, and selective reporting. Sample sizes varied considerably (20-3902 participants), and most used cross-sectional designs, precluding longitudinal assessment. Importantly, only 47% of studies focused on clinical efficacy testing, with most LLM-based studies (77%) remaining in early validation phases [15]. Technological advancement has progressed faster than the multistage validation processes necessary for robust clinical evidence, resulting in limited data on long-term effectiveness and real-world implementation. Heterogeneity in reported metrics and a lack of standardized evaluation frameworks complicated cross-study comparisons and precluded meta-analysis for most outcomes.

### Clinical Implications

The evidence suggests several potential applications for AI-driven assessment tools. First, chatbots administering standardized instruments demonstrate equivalence with traditional modes, offering opportunities for remote screening, automated triage, and population scrutiny [35,42]. This could help address treatment gaps in underserved areas and reduce waiting times for initial assessment. Second, LLMs show promise as clinical decision support tools for extracting structured information from clinical narratives [46], identifying at-risk individuals requiring urgent evaluation [27], and augmenting cognitive assessment in resource-limited settings [12].

However, safe implementation requires clear clinical protocols, professional oversight, and integration with existing health care workflows. AI should function as a complementary tool that enhances rather than replaces clinical judgment. Hybrid models combining automated screening with human review for positive or uncertain cases may optimize sensitivity while maintaining specificity. EHR integration is essential for continuity of care, but it requires attention to data security, patient consent, and algorithmic transparency. Critically, the absence of longitudinal studies limits conclusions about sustained accuracy, calibration drift, or adaptation to changing diagnostic criteria.

### Algorithmic Bias and Health Equity

A major concern emerging from this review is the potential for AI systems to perpetuate or amplify health disparities. Empirical work shows that performance and risk estimates can vary across demographic subgroups, underscoring the importance of bias evaluation and mitigation [28,30]. Adler et al [30] demonstrated that sensed-behavioral patterns predictive of depression vary substantially across demographic subgroups, with AI tools incorrectly ranking individuals with depression from certain groups as lower risk than healthier individuals from other groups. Qualitative analyses reveal that race-explicit or race-implied patient information frequently results in inferior treatment recommendations from LLMs [7], and natural language processing models exhibit measurable biases related to religion, race, gender, nationality, sexuality, and age [31,32].

Moreover, acceptability findings suggest differential engagement by age, education, and technological familiarity, with older adults and those with lower digital literacy reporting lower acceptability [48], potentially expanding access gaps for vulnerable populations. Addressing these inequities requires diverse and representative training datasets, rigorous bias testing across protected characteristics, transparency in algorithmic decision-making, human oversight mechanisms, and continuous monitoring of real-world performance stratified by demographic factors.

### Ethical Considerations and Implementation Challenges

Beyond bias, several ethical concerns require attention. The “black box” nature of many LLMs raises questions about accountability when errors occur [49]. Clear governance frameworks delineating roles and liabilities are essential. Informed consent presents unique challenges, as patients must understand when AI is involved in their assessment, how their data will be used, and the limitations of algorithmic predictions. Data privacy and security are paramount, given the sensitivity of mental health information. The risk of overreliance on automated systems could lead to deskilling of clinicians or premature diagnostic closure. AI should augment, not replace, comprehensive clinical assessment that considers contextual factors, patient preferences, and therapeutic relationships. Implementation also faces practical barriers, including EHR integration, evolving regulatory frameworks, underdeveloped reimbursement mechanisms, and the need for clinician training.

### Future Directions

Several critical gaps require attention. First, prospective longitudinal studies are needed to evaluate sustained performance, calibration over time, and clinical utility in real-world settings. Second, comparative effectiveness research should directly compare AI-driven assessment against standard care in pragmatic trials, measuring diagnostic accuracy, access, efficiency, and clinical outcomes. Third, research must systematically evaluate performance across diverse populations with prespecified, adequately powered subgroup analyses. Fourth, studies should explore optimal human-AI collaboration models and the required clinician training. Fifth, research on algorithmic fairness and bias mitigation strategies is essential. Sixth, implementation science research should identify facilitators, barriers, and sustainment strategies. Finally, regulatory science research is needed to establish appropriate evaluation frameworks and postmarket surveillance requirements.

### Implications for Policy and Practice

Health care systems considering AI adoption should prioritize infrastructure for secure data management, clinical protocols specifying appropriate use cases and oversight requirements, clinician training programs, bias monitoring mechanisms, and patient engagement. Regulatory bodies should develop clear approval pathways requiring evidence of clinical validity, algorithmic transparency, bias testing, and ongoing surveillance. Professional organizations should establish clinical practice guidelines for AI-assisted assessment, addressing appropriate use cases, limitations, documentation requirements, and ethical obligations. Researchers and developers should prioritize open science practices, including sharing algorithms and evaluation frameworks where ethically permissible. Policymakers should ensure that AI deployment does not exacerbate existing disparities and that vulnerable populations maintain access to human-delivered care.

### Conclusions

AI-driven conversational agents demonstrate clinically relevant performance in mental health screening and assessment, with potential to expand access and to support clinical decision-making. However, realizing this potential requires addressing substantial methodological, ethical, and practical challenges. The evidence base remains limited by heterogeneity, short-term follow-up, and moderate risk of bias. Algorithmic bias poses serious threats to health equity that demand proactive mitigation. Implementation requires careful attention to clinical integration, human oversight, transparency, and patient engagement. The goal should not be to replace human clinicians but to develop complementary tools that enhance efficiency, access, and quality while preserving the essential human elements of empathy, contextual understanding, and ethical judgment. A collaborative model integrating AI capabilities with human expertise may offer a path toward more accessible, timely, and equitable mental health assessment. However, this vision requires sustained research, thoughtful regulation, and a commitment to equity as technological capabilities continue to evolve.

## Supplementary material

10.2196/93672 Multimedia Appendix 1 Search equations for PubMed and other databases.

10.2196/93672 Multimedia Appendix 2 Risk of bias assessment.

10.2196/93672 Checklist 1 PRISMA checklist.

## Abbreviations

AIM: Acceptability of Intervention Measure
AUC: area under the curve
EHR: electronic health record
fMRI: functional magnetic resonance imaging
GAD-7: Generalized Anxiety Disorder-7
JBI: Joanna Briggs Institute
LLM: large language model
ML: machine learning
MMAT: Mixed Methods Appraisal Tool
PHQ-9: Patient Health Questionnaire-9
PIO: Population, Intervention, Outcome
PRISMA: Preferred Reporting Items for Systematic Reviews and Meta-Analyses
PROSPERO: International Prospective Register of Systematic Reviews
PTSD: posttraumatic stress disorder
QUADAS-2: Quality Assessment of Diagnostic Accuracy Studies-2
RCT: randomized controlled trial
RoB 2: Risk of Bias 2
SIRI-2: Suicidal Ideation Response Inventory–Revised
SPIN: Social Phobia Inventory
XGBoost: Extreme Gradient Boosting
