# Supplementary material for: Effectiveness of Chatbots in Mental Health Screening and Assessment: Systematic Review
Source: JMIR Ment Health. 2026 Oct 6;13:e93672. doi: 10.2196/93672 (PMC13641319; doi:10.2196/93672)
Supplement: Supplementary file 1 [file mental-v13-e93672-s001.docx]

**Pubmed**:

(

"Mental Disorders"[Mesh]

OR "Anxiety Disorders"[Mesh]

OR "Depressive Disorder"[Mesh]

OR "Mood Disorders"[Mesh]

OR "Bipolar Disorder"[Mesh]

OR "Substance-Related Disorders"[Mesh]

OR "mental health"[tiab]

OR "mental disorder*"[tiab]

OR "psychiatric disorder*"[tiab]

OR psychiatr*[tiab]

OR depress*[tiab]

OR anxi*[tiab]

OR bipolar[tiab]

OR "mood disorder*"[tiab]

OR "substance use disorder*"[tiab]

)

AND

(

chatbot*[tiab]

OR "chat bot*"[tiab]

OR "conversational agent*"[tiab]

OR "conversational AI"[tiab]

OR "virtual agent*"[tiab]

OR "virtual assistant*"[tiab]

OR "dialogue system*"[tiab]

OR "dialog system*"[tiab]

OR "large language model*"[tiab]

OR LLM[tiab]

OR LLMs[tiab]

OR ChatGPT[tiab]

OR GPT-3[tiab]

OR "GPT-3.5"[tiab]

OR GPT-4[tiab]

OR GPT-4o[tiab]

OR Claude[tiab]

OR Gemini[tiab]

OR Llama[tiab]

OR DeepSeek[tiab]

)

AND

(

"Psychological Tests"[Mesh]

OR "Psychometrics"[Mesh]

OR "Mass Screening"[Mesh]

OR "Diagnosis"[Mesh]

OR "Decision Support Systems, Clinical"[Mesh]

OR "psychological assessment"[tiab]

OR "psychological evaluation"[tiab]

OR "psychiatric assessment"[tiab]

OR "mental health assessment"[tiab]

OR screen*[tiab]

OR assess*[tiab]

OR diagnos*[tiab]

OR detect*[tiab]

OR triag*[tiab]

OR psychometr*[tiab]

OR questionnaire*[tiab]

OR "clinical interview*"[tiab]

OR "risk assessment"[tiab]

OR "clinical decision support"[tiab]

OR validat*[tiab]

OR accurac*[tiab]

OR reliab*[tiab]

OR acceptab*[tiab]

OR usability[tiab]

OR feasibility[tiab]

)

AND

(

adult[Mesh] OR adult*[tiab]

)

AND

(

english[lang] OR spanish[lang]

)

AND

(

2019/01/01:2026/04/12[dp]

)

NOT

(

child[Mesh] NOT adult[Mesh]

)

**EMBASE:**

(

'mental disease'/exp

OR 'anxiety disorder'/exp

OR 'depression'/exp

OR 'mood disorder'/exp

OR 'bipolar disorder'/exp

OR 'substance use disorder'/exp

OR 'mental health':ti,ab

OR 'mental disorder*':ti,ab

OR 'psychiatric disorder*':ti,ab

OR psychiatr*:ti,ab

OR depress*:ti,ab

OR anxi*:ti,ab

OR bipolar:ti,ab

OR 'mood disorder*':ti,ab

OR 'substance use disorder*':ti,ab

)

AND

(

chatbot*:ti,ab

OR 'chat bot*':ti,ab

OR 'conversational agent*':ti,ab

OR 'conversational AI':ti,ab

OR 'virtual agent*':ti,ab

OR 'virtual assistant*':ti,ab

OR 'dialogue system*':ti,ab

OR 'dialog system*':ti,ab

OR 'large language model*':ti,ab

OR LLM:ti,ab

OR LLMs:ti,ab

OR ChatGPT:ti,ab

OR 'GPT-3':ti,ab

OR 'GPT-3.5':ti,ab

OR 'GPT-4':ti,ab

OR 'GPT-4o':ti,ab

OR Claude:ti,ab

OR Gemini:ti,ab

OR Llama:ti,ab

OR DeepSeek:ti,ab

)

AND

(

'psychological test'/exp

OR 'psychometry'/exp

OR 'mass screening'/exp

OR 'diagnosis'/exp

OR 'clinical decision support system'/exp

OR 'psychological assessment':ti,ab

OR 'psychological evaluation':ti,ab

OR 'psychiatric assessment':ti,ab

OR 'mental health assessment':ti,ab

OR screen*:ti,ab

OR assess*:ti,ab

OR diagnos*:ti,ab

OR detect*:ti,ab

OR triag*:ti,ab

OR psychometr*:ti,ab

OR questionnaire*:ti,ab

OR 'clinical interview*':ti,ab

OR 'risk assessment':ti,ab

OR 'clinical decision support':ti,ab

OR validat*:ti,ab

OR accurac*:ti,ab

OR reliab*:ti,ab

OR acceptab*:ti,ab

OR usability:ti,ab

OR feasibility:ti,ab

)

AND

(

'adult'/exp

OR adult*:ti,ab

)

AND

(

[english]/lim

OR [spanish]/lim

)

AND

(

[2019-2026]/py

)

NOT

(

'child'/exp NOT 'adult'/exp

)

**SCOPUS**: TITLE-ABS-KEY(

(

"mental health" OR "mental disorder*" OR "psychiatric disorder*" OR psychiatr*

OR depress* OR anxi* OR bipolar OR "mood disorder*" OR "substance use disorder*"

)

AND

(

chatbot* OR "chat bot*" OR "conversational agent*" OR "conversational AI"

OR "virtual agent*" OR "virtual assistant*" OR "dialogue system*" OR "dialog system*"

OR "large language model*" OR LLM OR LLMs OR ChatGPT

OR "GPT-3" OR "GPT-3.5" OR "GPT-4" OR "GPT-4o"

OR Claude OR Gemini OR Llama OR DeepSeek

)

AND

(

"psychological assessment" OR "psychological evaluation"

OR "psychiatric assessment" OR "mental health assessment"

OR screen* OR assess* OR diagnos* OR detect* OR triag*

OR psychometr* OR questionnaire* OR "clinical interview*"

OR "risk assessment" OR "clinical decision support"

OR validat* OR accurac* OR reliab*

OR acceptab* OR usability OR feasibility

)

AND

(

adult OR adults

)

)

AND

(

LIMIT-TO(LANGUAGE, "English") OR LIMIT-TO(LANGUAGE, "Spanish")

)

AND

PUBYEAR > 2018

AND

PUBYEAR < 2027

AND NOT TITLE-ABS-KEY(

child* OR adolescen* OR pediatric* OR paediatric*

)

**WOS**

TS=(

(

"mental health" OR "mental disorder*" OR "psychiatric disorder*" OR psychiatr*

OR depress* OR anxi* OR bipolar OR "mood disorder*" OR "substance use disorder*"

)

AND

(

chatbot* OR "chat bot*" OR "conversational agent*" OR "conversational AI"

OR "virtual agent*" OR "virtual assistant*" OR "dialogue system*" OR "dialog system*"

OR "large language model*" OR LLM OR LLMs OR ChatGPT

OR "GPT-3" OR "GPT-3.5" OR "GPT-4" OR "GPT-4o"

OR Claude OR Gemini OR Llama OR DeepSeek

)

AND

(

"psychological assessment" OR "psychological evaluation"

OR "psychiatric assessment" OR "mental health assessment"

OR screen* OR assess* OR diagnos* OR detect* OR triag*

OR psychometr* OR questionnaire* OR "clinical interview*"

OR "risk assessment" OR "clinical decision support"

OR validat* OR accurac* OR reliab*

OR acceptab* OR usability OR feasibility

)

AND

(

adult OR adults

)

)

NOT TS=(child* OR adolescen* OR pediatric* OR paediatric*)

**PSYINFO**

(("Mental Disorders" OR "Anxiety Disorders" OR "Major Depression" OR "Mood Disorders" OR "Bipolar Disorder" OR "Substance Use Disorder") OR ("mental health" OR "mental disorder*" OR psychiatr* OR depress* OR anxi*)) AND (chatbot* OR "chat bot*" OR "conversational agent*" OR "virtual agent*" OR "virtual assistant*" OR "dialogue system*" OR "large language model*" OR LLM OR ChatGPT OR "GPT-4") AND ("psychological assessment" OR "mental health assessment" OR screen* OR assess* OR diagnos*)

The PubMed search was conducted using a combination of MeSH terms and free-text keywords related to mental health conditions, conversational AI/chatbots, and screening/assessment. The full search strategy included controlled vocabulary (e.g., "Mental Disorders"[MeSH], "Artificial Intelligence"[MeSH], "Psychometrics"[MeSH]) and text words (e.g., chatbot*, "large language model*", screen*, assess*). The search was limited to articles published between January 1, 2019 and April 12, 2026, in English or Spanish.

Records retrieved: 447.

The Embase search was performed using EMTREE terms and free-text keywords covering mental health conditions, conversational agents/chatbots, and screening or assessment processes. Both controlled vocabulary (e.g., 'mental disease'/exp, 'psychological test'/exp) and text terms (e.g., chatbot*, "virtual agent*", assess*) were used. Limits were applied to publication years 2019–2026 and to English and Spanish language articles.

Records retrieved: [añadir número].

The Scopus search was conducted using title, abstract, and keyword fields (TITLE-ABS-KEY), combining terms related to mental health, conversational AI/chatbots, and screening or assessment. The search included synonyms and variations (e.g., chatbot*, "conversational agent*", screen*, diagnos*). Results were limited to publications between 2019 and 2026 and to English and Spanish languages.

Records retrieved: 576.

The Web of Science Core Collection was searched using the Topic field (TS), which includes title, abstract, and keywords. The strategy combined terms related to mental health, conversational AI/chatbots, and screening or assessment. Filters for publication years (2019–2026) and language (English and Spanish) were applied through the database interface.

Records retrieved: 190.

The APA PsycInfo search was conducted using both controlled vocabulary (APA Thesaurus terms) and free-text keywords across three domains: mental health conditions, conversational AI/chatbots, and screening/assessment. The final query was executed in command-line mode to ensure correct Boolean logic and grouping of terms. Results were limited to publications from 2019 to 2026 in English and Spanish.

Records retrieved: 364.

The search strategies were designed to maximise sensitivity and minimise the risk of missing relevant studies, following PRISMA-S recommendations. Full database-specific search strategies are provided in the supplementary materials.

Broad terms such as “Artificial Intelligence” were not used as primary search terms to avoid excessive retrieval of irrelevant studies not involving conversational agents.

The search strategy combined controlled vocabulary (MeSH, EMTREE, and APA Thesaurus terms) with free-text keywords to maximise sensitivity and minimise the risk of missing relevant studies, following PRISMA-S recommendations.

The search strategies were iteratively refined with the aim of balancing sensitivity and specificity, ensuring comprehensive coverage of conversational AI applications in mental health screening and assessment.
