# Supplementary material for: Effectiveness of Chatbots in Mental Health Screening and Assessment: Systematic Review
Source: JMIR Ment Health. 2026 Oct 6;13:e93672. doi: 10.2196/93672 (PMC13641319; doi:10.2196/93672)
Supplement: Supplementary file 2 [file mental-v13-e93672-s002.docx]

**Supplementary Material 1. Risk-of-bias assessment**

This supplementary material provides the complete risk-of-bias assessment of the studies included in the systematic review. Studies were assessed using design-specific tools according to their methodological design: QUADAS-2 for diagnostic accuracy and validation studies, the JBI Critical Appraisal Checklist for Analytical Cross-Sectional Studies for analytical cross-sectional studies, the JBI Checklist for Quasi-Experimental Studies for quasi-experimental, pilot, pre–post, feasibility, or non-randomized intervention studies, and the JBI Checklist for Cohort Studies for cohort studies.

Overall judgements were harmonized as low risk of bias, moderate risk of bias, or high risk of bias. Traffic-light plots and weighted bar plots were generated to summarize domain-level judgements.

Figures were generated using robvis/RoBVIS with a colour-blind friendly palette.

**Supplementary Table S1. Full risk-of-bias assessment of included studies**

This table summarizes the methodological design, risk-of-bias tool, overall judgement, and main reasons supporting each judgement for each included study.

| **Study** | **Study design** | **Risk-of-bias tool** | **Overall judgement** | **Main reasons** |
| --- | --- | --- | --- | --- |
| Anmella et al. (2023) | Sequential uncontrolled feasibility and potential-effectiveness pilot study | JBI Checklist for Quasi-Experimental Studies | High risk of bias | No control group, small sample, high attrition, and limited causal inference. |
| Auriacombe et al. (2021) | App-based observational cross-sectional study with short follow-up diary and acceptability assessment | JBI Checklist for Analytical Cross-Sectional Studies | Moderate risk of bias | Large sample, but self-selected users, self-reported outcomes, limited follow-up, and no clinical reference standard. |
| Chanteclair et al. (2025) | Preliminary cross-sectional acceptability study in geriatric inpatients | JBI Checklist for Analytical Cross-Sectional Studies | Moderate risk of bias | Small single-centre sample, supervised one-time use, selective exclusions, and no full adherence assessment. |
| Dosovitsky et al. (2021) | Cross-sectional psychometric validation study of a chatbot-administered PHQ-9 | JBI Checklist for Analytical Cross-Sectional Studies | Moderate risk of bias | Large sample and high completion rate, but self-selected users, female predominance, and no clinical reference standard. |
| Du et al. (2024) | Retrospective diagnostic accuracy/model validation study using EHR clinical notes | QUADAS-2 | Moderate risk of bias | Single-system EHR data, imperfect reference standard, section-level validation, and no fully external validation. |
| Hemrungrojn et al. (2024) | Cross-sectional diagnostic accuracy and concordance study | QUADAS-2 | Moderate risk of bias | Purposive hospital sample, imperfect reference standard, unclear blinding, and limited generalisability. |
| Hur et al. (2024) | Prospective online cohort study with short-term symptom prediction | JBI Checklist for Cohort Studies | Moderate risk of bias | Self-selected online sample, short follow-up, self-reported PHQ-9 outcome, and no clinical diagnostic reference standard. |
| Kaywan et al. (2023) | Non-clinical cross-sectional feasibility and usability study | JBI Checklist for Analytical Cross-Sectional Studies | High risk of bias | Small convenience sample, high dropout, no clinical comparator, no longitudinal assessment, and manual scoring. |
| Lau et al. (2023) | Secondary model development and validation study using clinical interview transcripts | JBI Checklist for Analytical Cross-Sectional Studies | Moderate risk of bias | Small benchmark dataset, PHQ-8 proxy outcome, no external validation, and limited clinical generalisability. |
| Lee et al. (2024) | Retrospective comparative diagnostic and prognostic accuracy study | QUADAS-2 | Moderate to high risk of bias | Enriched retrospective sample, self-reported outcome, limited predictors, no external validation, and industry conflict. |
| Li (2025) | Multimodal diagnostic model development and validation study | QUADAS-2 | Moderate to high risk of bias | Small single-centre sample, unclear reference standard, limited reporting, and restricted generalisability. |
| Martinez-Romo et al. (2025) | Retrospective diagnostic model development and validation study using EHR text | QUADAS-2 | Moderate risk of bias | Single-hospital test corpus, EHR-based reference labels, class imbalance, and no external validation. |
| McCoy & Perlis (2024) | Retrospective EHR-based cohort study using LLM-derived dimensional psychopathology scores | JBI Checklist for Cohort Studies | Moderate risk of bias | Single-centre EHR cohort, no gold standard, documentation bias, and limited outcome capture. |
| Ohse et al. (2024) | Cross-sectional diagnostic concordance study using interview transcripts and SPIN scores | QUADAS-2 | Moderate to high risk of bias | Small convenience sample, self-report reference standard, translated transcripts, and no clinical diagnosis or external validation. |
| Otsuka et al. (2023) | Retrospective diagnostic model development and validation study using discharge-summary HPI text | QUADAS-2 | Moderate to high risk of bias | Single-centre retrospective data, imperfect reference standard, possible information leakage, class imbalance, and no external validation. |
| Palominos et al. (2024) | Cross-sectional computational speech analysis study | JBI Checklist for Analytical Cross-Sectional Studies | Moderate risk of bias | Modest clinical sample, task-specific speech elicitation, education imbalance, residual confounding, and no external validation. |
| Pugh et al. (2024) | Cross-sectional computational validation study using transcribed speech samples | JBI Checklist for Analytical Cross-Sectional Studies | Moderate risk of bias | Small legacy sample, expert-rating reference, manual transcription, model inconsistency, and no external validation. |
| Santa-Cruz et al. (2022) | Uncontrolled population-based digital mental health intervention with pre-post follow-up | JBI Checklist for Quasi-Experimental Studies | High risk of bias | No control group, self-selected participants, substantial loss to follow-up, and self-reported outcomes. |
| Schick et al. (2022) | Randomized laboratory-based experimental validation study of chatbot assessment modes | JBI Checklist for Quasi-Experimental Studies | High risk of bias | Student sample, non-clinical setting, short washout, possible memory effects, and limited external validity. |
| Shin et al. (2024) | Diagnostic model development and validation study using user-generated diary text | QUADAS-2 | Moderate to high risk of bias | Small self-selected sample, imbalanced classes, imperfect reference standard, no external validation, and industry conflict. |
| So et al. (2024) | Pilot computational validation study using psychiatric interview transcripts | JBI Checklist for Analytical Cross-Sectional Studies | Moderate to high risk of bias | Very small in-house sample, narrow clinical population, transcript-based analysis, expert-label dependence, and no external validation. |
| Wiest et al. (2024) | Retrospective diagnostic accuracy study using psychiatric EHR admission notes | QUADAS-2 | Moderate risk of bias | Small single-centre sample, binary expert reference standard, prompt variability, and no external validation. |
| Kosyluk et al. (2024) | Cross-sectional acceptability and mental health screening study using a chatbot-administered PHQ-9 | JBI Checklist for Analytical Cross-Sectional Studies | Moderate risk of bias | High completion rate and clear screening procedure, but self-selected sample, self-reported outcomes, limited control of confounding, and no clinical reference standard. |

**Supplementary Figure S1. QUADAS-2 traffic-light plot**

Traffic-light plot of diagnostic accuracy and validation studies assessed using QUADAS-2.

**Supplementary Figure S2. QUADAS-2 weighted bar plot**

Weighted bar plot summarizing the distribution of risk-of-bias judgements across QUADAS-2 domains.

**Supplementary Figure S3. JBI Analytical Cross-Sectional Studies traffic-light plot**

Traffic-light plot of analytical cross-sectional studies assessed using the JBI Critical Appraisal Checklist for Analytical Cross-Sectional Studies.

**Supplementary Figure S4. JBI Analytical Cross-Sectional Studies weighted bar plot**

Weighted bar plot summarizing the distribution of risk-of-bias judgements across JBI Analytical Cross-Sectional domains.

**Supplementary Figure S5. JBI Quasi-Experimental Studies traffic-light plot**

Traffic-light plot of quasi-experimental, pilot, pre–post, feasibility, or non-randomized intervention studies assessed using the JBI Checklist for Quasi-Experimental Studies.

**Supplementary Figure S6. JBI Quasi-Experimental Studies weighted bar plot**

Weighted bar plot summarizing the distribution of risk-of-bias judgements across JBI Quasi-Experimental domains.

**Supplementary Figure S7. JBI Cohort Studies traffic-light plot**

Traffic-light plot of cohort studies assessed using the JBI Checklist for Cohort Studies.

**Supplementary Figure S8. JBI Cohort Studies weighted bar plot**

Weighted bar plot summarizing the distribution of risk-of-bias judgements across JBI Cohort Studies domains.
