# Supplementary material for: Effectiveness of Chatbots in Mental Health Screening and Assessment: Systematic Review
Source: JMIR Ment Health. 2026 Oct 6;13:e93672. doi: 10.2196/93672 (PMC13641319; doi:10.2196/93672)
Supplement: Supplementary file 3 [file mental-v13-e93672-s003.docx]

# **PRISMA checklist**

# **PRISMA 2020 Main Checklist**

| **Topic** | **No.** | **Item** | **Reported?** |
| --- | --- | --- | --- |
| **TITLE** |  |  |  |
| **Title** | 1 | Identify the report as a systematic review. | Yes |
| **BACKGROUND** |  |  |  |
| **Objectives** | 2 | Provide an explicit statement of the main objective(s) or question(s) the review addresses. | Yes |
| **METHODS** |  |  |  |
| **Eligibility criteria** | 3 | Specify the inclusion and exclusion criteria for the review. | Yes |
| **Information sources** | 4 | Specify the information sources (e.g. databases, registers) used to identify studies and the date when each was last searched. | Yes |
| **Risk of bias** | 5 | Specify the methods used to assess risk of bias in the included studies. | Yes |
| **Synthesis of results** | 6 | Specify the methods used to present and synthesize results. | Yes |
| **RESULTS** |  |  |  |
| **Included studies** | 7 | Give the total number of included studies and participants and summarise relevant characteristics of studies. | Yes |
| **Synthesis of results** | 8 | Present results for main outcomes, preferably indicating the number of included studies and participants for each. If meta-analysis was done, report the summary estimate and confidence/credible interval. If comparing groups, indicate the direction of the effect (i.e. which group is favoured). | Yes |
| **DISCUSSION** |  |  |  |
| **Limitations of evidence** | 9 | Provide a brief summary of the limitations of the evidence included in the review (e.g. study risk of bias, inconsistency and imprecision). | Yes |
| **Interpretation** | 10 | Provide a general interpretation of the results and important implications. | Yes |
| **OTHER** |  |  |  |
| **Funding** | 11 | Specify the primary source of funding for the review. | Yes |
| **Registration** | 12 | Provide the register name and registration number. | Yes |

| **Topic** | **No.** | **Item** | **Location where item is reported** |
| --- | --- | --- | --- |
| **TITLE** |  |  |  |
| **Title** | 1 | Identify the report as a systematic review. | Title (Page 1). Explicitly identified as "A Systematic Review". |
| **ABSTRACT** |  |  |  |
| **Abstract** | 2 | See the PRISMA 2020 for Abstracts checklist |  |
| **INTRODUCTION** |  |  |  |
| **Rationale** | 3 | Describe the rationale for the review in the context of existing knowledge. | Section 1. Introduction (Pages 1-3). Discusses global mental health burden, the treatment gap, and the evolution of AI/LLMs since 2023. |
| **Objectives** | 4 | Provide an explicit statement of the objective(s) or question(s) the review addresses. | Abstract (Page 1) and Section 1. Introduction (Page 3). Aimed at evaluating effectiveness, accuracy, reliability, and acceptability. |
| **METHODS** |  |  |  |
| **Eligibility criteria** | 5 | Specify the inclusion and exclusion criteria for the review and how studies were grouped for the syntheses. | Section 2.2. Eligibility Criteria (Pages 5). Details population (adults), intervention (chatbots/AI), and study designs. Grouping is detailed in Section 3.3 (Page 6). |
| **Information sources** | 6 | Specify all databases, registers, websites, organisations, reference lists and other sources searched or consulted to identify studies. Specify the date when each source was last searched or consulted. | Section 2.1 & 2.3 (Pages 5-6). Databases: PubMed/MEDLINE, Embase, PsycINFO, Scopus, and Web of Science. Last search: April 2026. |
| **Search strategy** | 7 | Present the full search strategies for all databases, registers and websites, including any filters and limits used. | Section 2.1 (Page 4). Includes PubMed example; full strategies for all databases are in Supplementary File 2. |
| **Selection process** | 8 | Specify the methods used to decide whether a study met the inclusion criteria of the review, including how many reviewers screened each record and each report retrieved, whether they worked independently, and if applicable, details of automation tools used in the process. | Section 2.3. Study Selection (Page 5). Three independent reviewers used Rayyan; discrepancies were resolved by a fourth reviewer. |
| **Data collection process** | 9 | Specify the methods used to collect data from reports, including how many reviewers collected data from each report, whether they worked independently, any processes for obtaining or confirming data from study investigators, and if applicable, details of automation tools used in the process. | Section 2.4 (Page 6). Two independent reviewers used a standardized template; a third reviewer resolved discrepancies. |
| **Data items** | 10a | List and define all outcomes for which data were sought. Specify whether all results that were compatible with each outcome domain in each study were sought (e.g. for all measures, time points, analyses), and if not, the methods used to decide which results to collect. | Section 2.1 (Page 4) and Section 2.3 (Page 5). Outcomes sought include effectiveness, diagnostic accuracy (sensitivity, specificity, AUC, F1-score), reliability (ICC, Cronbach’s α), validity, acceptability, and feasibility. |
|  | 10b | List and define all other variables for which data were sought (e.g. participant and intervention characteristics, funding sources). Describe any assumptions made about any missing or unclear information. | Section 2.4 (Page 6). Collected variables: author, year, country, language, sample size, diagnosis/condition, chatbot model/architecture, interaction modality, and reference instruments. |
| **Study risk of bias assessment** | 11 | Specify the methods used to assess risk of bias in the included studies, including details of the tool(s) used, how many reviewers assessed each study and whether they worked independently, and if applicable, details of automation tools used in the process. | Section 2.4 (Page 6) and Section 3.4 (Page 22-23). Evaluated by two independent reviewers using QUADAS-2 (diagnostic) and JBI checklists (observational/qualitative). |
| **Effect measures** | 12 | Specify for each outcome the effect measure(s) (e.g. risk ratio, mean difference) used in the synthesis or presentation of results. | Section 2.4 (Page 6) and Section 3.2 (Page 7-18) and Section . Measures include Sensitivity (S), Specificity (E), Area Under the Curve (AUC), F1-score, accuracy, Intraclass Correlation (ICC), and Pearson’s correlation (r). |
| **Synthesis methods** | 13a | Describe the processes used to decide which studies were eligible for each synthesis (e.g. tabulating the study intervention characteristics and comparing against the planned groups for each synthesis (item 5)). | Section 3.3 (Page 19-21). Studies were grouped into three dimensions: screening applications, diagnostic classification, and prediction and prognostic applications. |
|  | 13b | Describe any methods required to prepare the data for presentation or synthesis, such as handling of missing summary statistics, or data conversions. | Section 3.3 (Page 11). Since most studies lacked confidence intervals (CI) or weights, reported point estimates were normalized to a 0–1 scale. |
|  | 13c | Describe any methods used to tabulate or visually display results of individual studies and syntheses. | Section 3.2 (Page 7-18). Results are presented in Table 1, forest plots for performance metrics (Figures 2-6), and stacked bar charts for risk of bias (supplementary file 1). |
|  | 13d | Describe any methods used to synthesize results and provide a rationale for the choice(s). If meta-analysis was performed, describe the model(s), method(s) to identify the presence and extent of statistical heterogeneity, and software package(s) used. | Section 3.1 (Page 7) and Section 3.3 (Page 19-21). A qualitative synthesis was performed. Meta-analysis was not conducted due to significant heterogeneity and lack of standardized reporting (missing CIs). |
|  | 13e | Describe any methods used to explore possible causes of heterogeneity among study results (e.g. subgroup analysis, meta-regression). | Section 3.3 (Page 19-21). Heterogeneity was explored through grouping by clinical condition. |
|  | 13f | Describe any sensitivity analyses conducted to assess robustness of the synthesized results. | Not reported. The sources mention that meta-analysis was precluded by data variability, thus specific sensitivity analyses for pooled effects were not applicable. |
| **Reporting bias assessment** | 14 | Describe any methods used to assess risk of bias due to missing results in a synthesis (arising from reporting biases). | Section 3.4 (Page 21-22) and Section 4.1 (Page 15). Reporting bias was assessed as a dimension within the methodological quality tools (Supplementary File 1). |
| **Certainty assessment** | 15 | Describe any methods used to assess certainty (or confidence) in the body of evidence for an outcome. | Section 3.4 (Page 13) and Section 4.3 (Page 24). Certainty was determined by the synthesis of methodological quality, consistency of metrics across conditions, and the presence of moderate risk of bias in critical dimensions. |
| **RESULTS** |  |  |  |
| **Study selection** | 16a | Describe the results of the search and selection process, from the number of records identified in the search to the number of studies included in the review, ideally using a flow diagram. | Section 3.1 (Page 7) and Figure 1 (PRISMA Diagram). Reports the flow from 2961 identified records to 23 included studies. |
|  | 16b | Cite studies that might appear to meet the inclusion criteria, but which were excluded, and explain why they were excluded. | Section 2.3 (Page 5-6). Lists full-text reports excluded due to non-eligible study type (n=10) or unrelated diagnosis (n=3). |
| **Study characteristics** | 17 | Cite each included study and present its characteristics. | Table 1 (Pages 8-15) and Section 3.3 (Page 19-21). Details author, year, country, N, condition, model, and reference instruments for all 23 studies. |
| **Risk of bias in studies** | 18 | Present assessments of risk of bias for each included study. | Section 3.4 (Pages 21-22) and Figures in Supplementary File 1. Shows assessments using QUADAS-2, and JBI, noting moderate risk in critical dimensions. |
| **Results of individual studies** | 19 | For all outcomes, present, for each study: (a) summary statistics for each group (where appropriate) and (b) an effect estimate and its precision (e.g. confidence/credible interval), ideally using structured tables or plots. | Table 1 (Pages 8-15) and Section 3.2 (Pages7-17). Presents performance metrics (AUC, F1, S, E, accuracy) for individual studies. |
| **Results of syntheses** | 20a | For each synthesis, briefly summarise the characteristics and risk of bias among contributing studies. | Section 3.4 (Page 22-23) and Section 4.3 (Page 24). Groups evidence by chatbot type and disorder, noting heterogeneous quality and moderate bias. |
|  | 20b | Present results of all statistical syntheses conducted. If meta-analysis was done, present for each the summary estimate and its precision (e.g. confidence/credible interval) and measures of statistical heterogeneity. If comparing groups, describe the direction of the effect. | Section 3.2 (Page 7-8, 15-18). Statistical synthesis was qualitative; meta-analysis was precluded due to data heterogeneity and missing confidence intervals. |
|  | 20c | Present results of all investigations of possible causes of heterogeneity among study results. | Section 3.3 (Pages 19-21). Explores heterogeneity through subgrouping by clinical functions. |
|  | 20d | Present results of all sensitivity analyses conducted to assess the robustness of the synthesized results. | Not reported. Sensitivity analyses for pooled effects were not performed as no formal meta-analysis was conducted. |
| **Reporting biases** | 21 | Present assessments of risk of bias due to missing results (arising from reporting biases) for each synthesis assessed. | Section 3.4 (Page 21-22) and Section 4.3 (Page 24). Selective reporting was identified as a moderate risk factor in critical domains during methodological assessment. |
| **Certainty of evidence** | 22 | Present assessments of certainty (or confidence) in the body of evidence for each outcome assessed. | Section 3.4 (Page 21-22) and Section 4.2 (Page 23-24) and Section 4.3. (24). Certainty is described as limited by methodological heterogeneity, small samples in 77% of LLM studies, and moderate risk of bias. |
| **DISCUSSION** |  |  |  |
| **Discussion** | 23a | Provide a general interpretation of the results in the context of other evidence. | Section 4. Discussion (Page 22-23). The review notes that chatbots achieve clinically relevant performance, extending prior reviews that focused primarily on therapeutic interventions rather than assessment and generative LLMs,. |
|  | 23b | Discuss any limitations of the evidence included in the review. | Section 4.2 (Page 23-24) and Section 4.3 (Page 24). Limitations include heterogeneous quality, short-term follow-up (lack of longitudinal data), moderate risk of bias in critical dimensions, and small sample sizes in most LLM-based studies,. |
|  | 23c | Discuss any limitations of the review processes used. | Section 4.2 (Page 23-24). The review acknowledges that the heterogeneity of reported metrics and lack of standardized evaluation frameworks precluded a meta-analysis for most outcomes. |
|  | 23d | Discuss implications of the results for practice, policy, and future research. | Sections 4.4, 4.5, 4.6, 4.7 (Pages 24-26). Implications for practice include remote screening and automated triage. Policy should address data management and approval pathways. Future research should focus on longitudinal clinical utility and algorithmic fairness. |
| **OTHER INFORMATION** |  |  |  |
| **Registration and protocol** | 24a | Provide registration information for the review, including register name and registration number, or state that the review was not registered. | Section 2. Protocol Registration (Page 4). Registered in PROSPERO with number CRD420251072392. |
|  | 24b | Indicate where the review protocol can be accessed, or state that a protocol was not prepared. | Section 2. Protocol Registration (Page 4). A link to the PROSPERO record is provided: https://www.crd.york.ac.uk/PROSPERO/display_record.php?RecordID=CRD420251072392. |
|  | 24c | Describe and explain any amendments to information provided at registration or in the protocol. | The review followed the predefined protocol and PRISMA 2020 guidelines. Checklist in Supplementary File 3. |
| **Support** | 25 | Describe sources of financial or non-financial support for the review, and the role of the funders or sponsors in the review. | Funding Statement (Page 31). |
| **Competing interests** | 26 | Declare any competing interests of review authors. | Conflicts of Interest (Page 31). |
| **Availability of data, code and other materials** | 27 | Report which of the following are publicly available and where they can be found: template data collection forms; data extracted from included studies; data used for all analyses; analytic code; any other materials used in the review. | Data Availability (Page 31) and Section 2.1 (Page 4). |

*From:* Page MJ, McKenzie JE, Bossuyt PM, Boutron I, Hoffmann TC, Mulrow CD, et al. The PRISMA 2020 statement: an updated guideline for reporting systematic reviews. MetaArXiv. 2020, September 14. DOI: 10.31222/osf.io/v7gm2. For more information, visit: [www.prisma-statement.org](http://www.prisma-statement.org)
